# Synthesis, crystal structure and Hirshfeld surface analysis of 2-(2,5-dioxo-4,4-di­phenyl­imidazolidin-1-yl)-*N*-(4-fluoro­phen­yl)acetamide (phenytoin analog)

**DOI:** 10.1107/S2056989026006201

**Published:** 2026-06-18

**Authors:** Hajar Ouabane, Chiara Massera, Walid Guerrab, Abdulsalam Alsubari, Joel T. Mague, Youssef Ramli

**Affiliations:** a Laboratory of Medicinal Chemistry Drug Sciences Research Center Faculty of Medicine and Pharmacy Mohammed V University in Rabat Morocco; b Dipartimento di Scienze Chimiche, della Vita e della Sostenibilità Ambientale, Università di Parma, Parco Area delle Scienze 17/A, 43124 Parma, Italy; c Life and Health Sciences Laboratory, Faculty of Medicine and Pharmacy, Abdelmalek Essaadi University, Tangier, Morocco; d Laboratory of Medicinal Chemistry, Faculty of Clinical Pharmacy, 21 September University, Yemen; e Department of Chemistry Tulane University New Orleans LA 70118 USA; Katholieke Universiteit Leuven, Belgium

**Keywords:** crystal structure, imidazolidine, acetamide, phenytoin analog, Hirshfeld surface

## Abstract

The title phenytoin analog 2-(2,5-dioxo-4,4-di­phenyl­imidazolidin-1-yl)-*N*-(4-fluoro­phen­yl)acetamide, C_23_H_18_FN_3_O_3_, was synthesized by alkyl­ation of phenytoin with 2-chloro-*N*-(4-fluoro­phen­yl)acetamide under phase-transfer catalysis conditions and crystallized as colorless blocks in the monoclinic space group *P*2_1_/*c*. In the mol­ecular structure, the imidazolidine ring shows a slight departure from planarity, adopting a twist conformation, while the two phenyl substituents are markedly inclined to the mean plane of the heterocyclic ring. The *N*-substituted 4-fluoro­phenyl­acetamide fragment projects away from the imidazolidine core, with its conformation partly consolidated by an intra­molecular C—H⋯O contact. In the crystal packing, pairs of inversion-related mol­ecules are linked through N—H⋯O hydrogen bonds to form dimers, which are further connected by additional N—H⋯O inter­actions into layers extending parallel to the *bc* plane. These layers stack along the *a*-axis direction through normal van der Waals contacts. Hirshfeld surface analysis was used to qu­antify the inter­molecular inter­actions, showing that H⋯H contacts give the largest contribution to the crystal packing, followed by C⋯H/H⋯C and O⋯H/H⋯O contacts, the latter being associated with the classical N—H⋯O hydrogen bonds. F⋯H/H⋯F inter­actions also contribute to the packing, mainly through contacts between adjacent mol­ecular layers.

## Abstract 2

In the title mol­ecule, the imidazolidine ring is slightly non-planar and the 4-fluoro­phenyl­acetamide substituent extends up and out from the mean plane of this ring. In the crystal, inversion-related N—H⋯O hydrogen bonds form dimers, which are connected into layers parallel to the *bc* plane by additional N—H⋯O hydrogen bonds. The layers pack along the *a*-axis direction with normal van der Waals contacts. A Hirshfeld surface analysis was performed.

## 1. Chemical context

**Figure 1:**
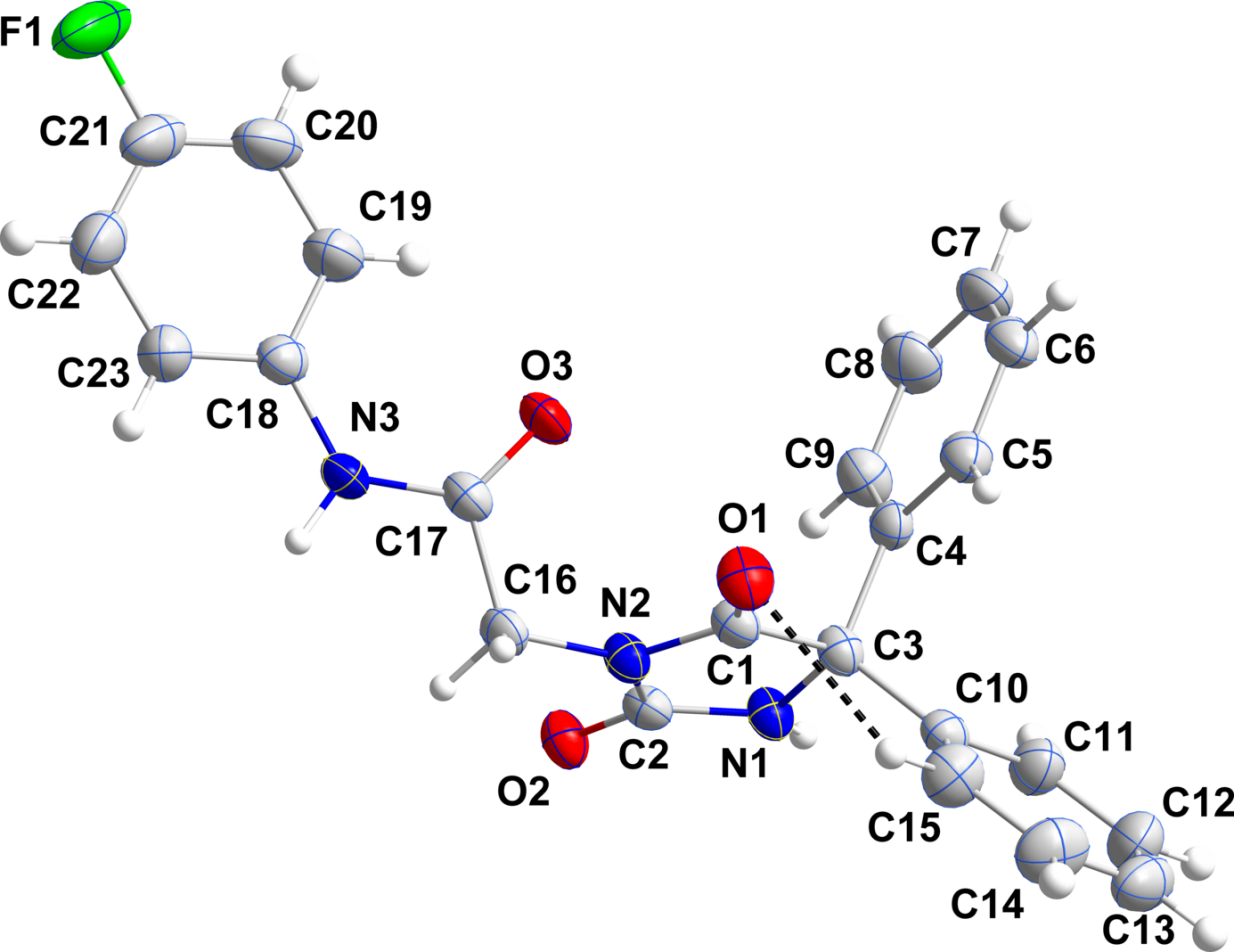
Perspective view of the title mol­ecule with the atom-labeling scheme and 50% probability ellipsoids. The intra­molecular hydrogen bond is depicted by a dashed line.

Imidazolidine is a five-membered, saturated, nonplanar, nona­romatic heterocycle with two nitro­gen atoms at the 1,3-positions. Imidazolidinone derivatives have attracted considerable attention due to their pharmacological and biological activities (Wadghane *et al.*, 2023 ▸). Imidazolidinones, including hydantoins, have been used as drugs such as phenytoin which is used in the treatment of epilepsy, anti­biotic nitro­furan­toin and anti­cancer drugs (apalutamide, nilutamide and enzalutamide), which are used to treat prostate cancer. Moreover, imidazolidinone derivatives exhibit various pharmacological activities such as anti­tumor (Elbadawi *et al.*, 2022 ▸), anti­depressant (Wessels *et al.*, 1980 ▸), anti­convulsant (Murasawa *et al.*, 2012 ▸), anti­viral (Khodair, 2002 ▸), anti­microbial (Kania *et al.*, 2022 ▸) and anti-inflammatory (El-Araby *et al.*, 2012 ▸). Similarly, a wide variety of compounds, including *N*-aryl­acetamides, have been reported to act as potential anti­diabetic agents (Moghimi *et al.*, 2020 ▸) and as anti­oxidant agents (Missioui *et al.*, 2021 ▸). In a continuation of our research on imidazolidinone derivatives (Guerrab *et al.*, 2025 ▸; El Moutaouakil Ala Allah *et al.*, 2025 ▸), we report herein the synthesis and crystal structure of the title compound C_23_H_18_FN_3_O_3_ (**3**) (Fig. 1 ▸) *via* an alkyl­ation of phenytoin with 2-chloro-*N*-(4-fluoro­phen­yl)acetamide under phase-transfer catalysis conditions. Hirshfeld surface analysis was performed to analyze the inter­molecular inter­actions.
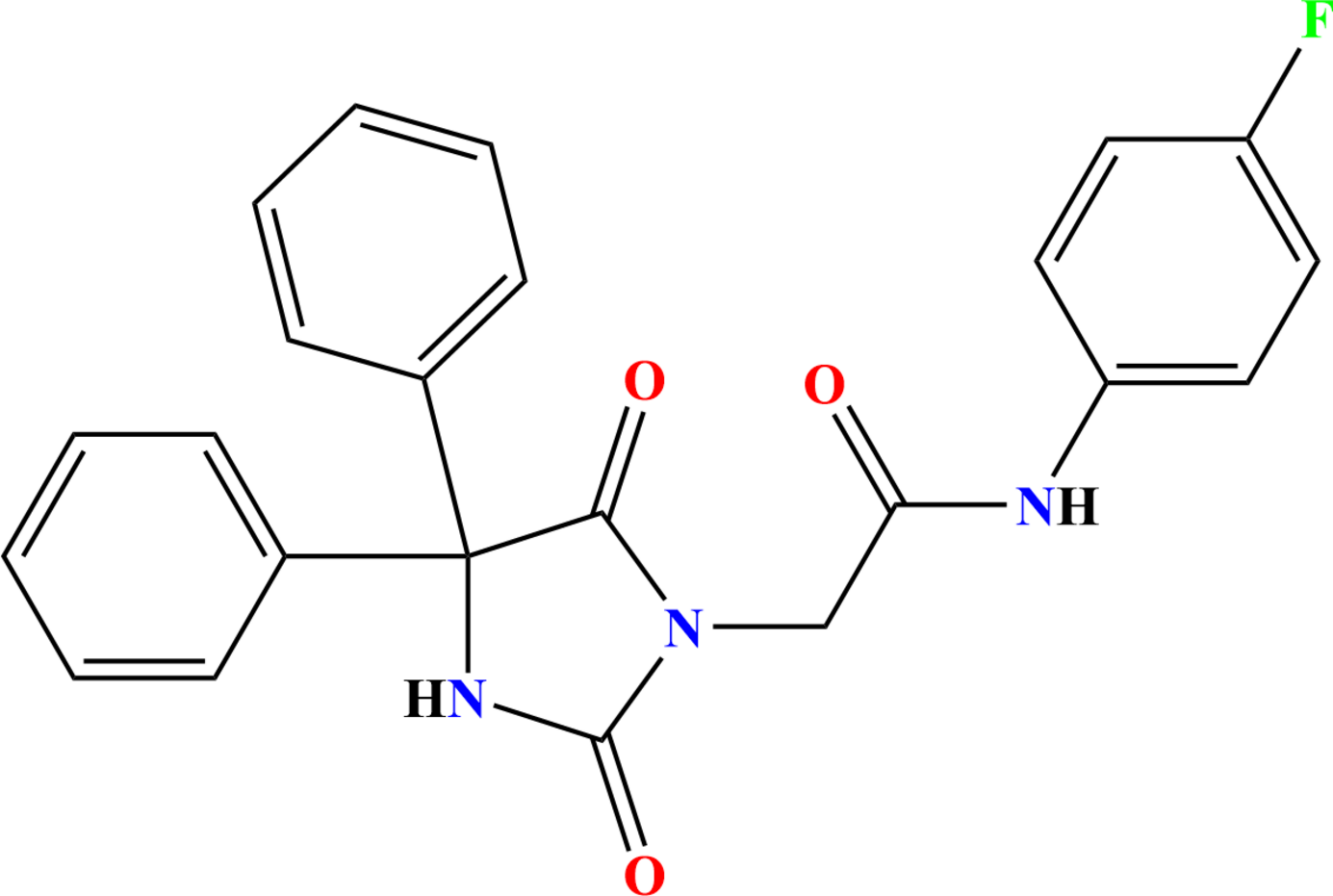


## 2. Structural commentary

In the title mol­ecule (**3**), the imidazolidine ring deviates modestly from planarity (r.m.s. deviation = 0.038 Å) and a puckering analysis (Cremer & Pople, 1975 ▸) gave the parameters *Q*(2) = 0.0852 (12) Å and φ(2) = 347.6 (8)°. The conformation of the ring is best described as a twist on C3—N1. The dihedral angles between the mean plane of the imidazolidine ring and those of the C4–C9 and the C10–C15 phenyl rings are 71.84 (7) and 54.44 (7)°, respectively. The substituent on N2 extends out from the mean plane of the imidazolidine ring. More precisely, the dihedral angle between the mean plane of the ring and that defined by N2, C16, C17 and O3 is 72.64 (8)° while the angle between the latter plane and that defined by O3, C17, N3 and C18 is 10.42 (8)°. Finally, the dihedral angle between the plane defined by O3, C17, N3 and C18 and the mean plane of the C18–C23 ring is 17.74 (8)°. The orientation of the C18–C23 ring with respect to the acetamide moiety is due, in part, to the intra­molecular C19—H19⋯O3 hydrogen bond (Table 1 ▸ and Fig. 1 ▸). The N1—C2 and N1—C3 bond distances are 1.338 (2) and 1.459 (1) Å, respectively, while the N2—C1 and N2—C2 distances are 1.372 (2) and 1.402 (2) Å, respectively, indicating involvement of the lone pairs of both nitro­gen atoms in N→C π-bonding. Clearly, this is clearly more observed for N1 than it is for N2.

## 3. Supra­molecular features

**Figure 2:**
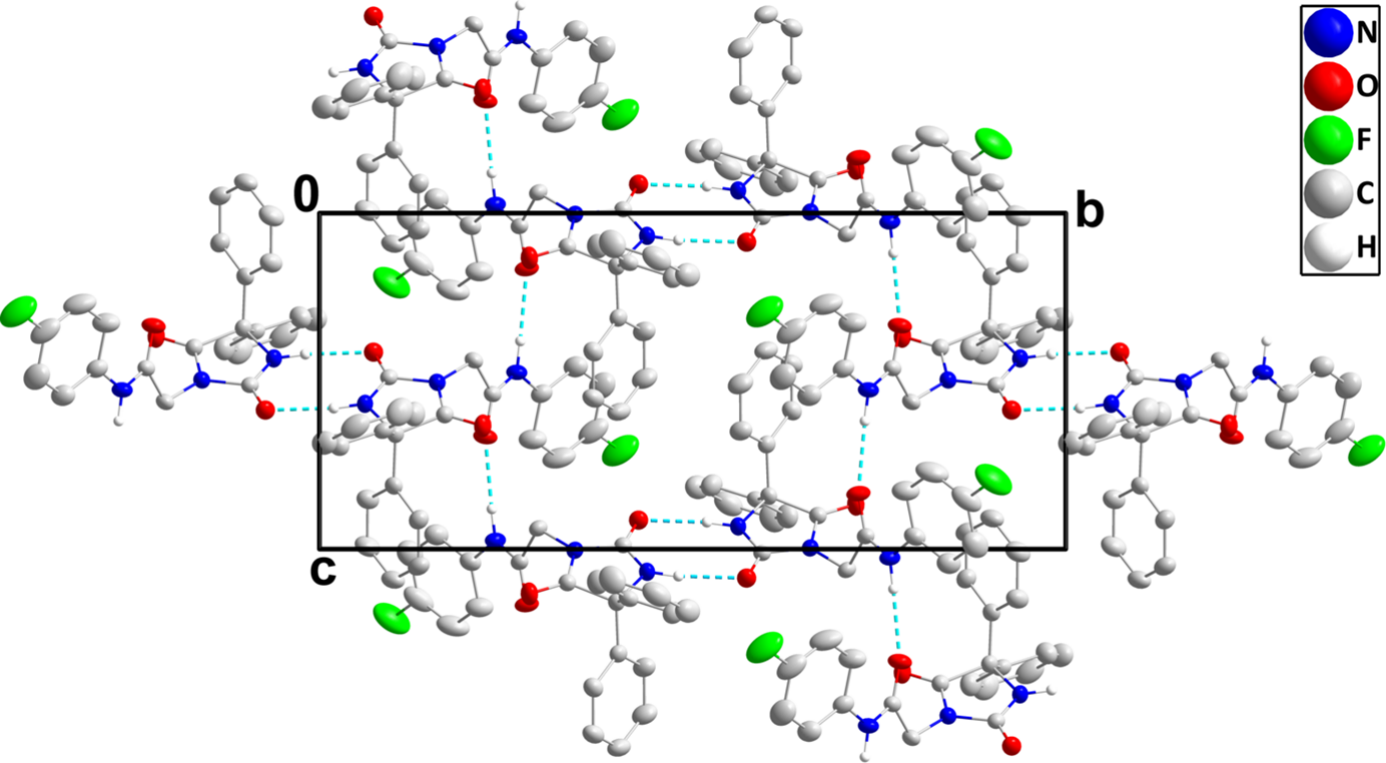
A portion of one layer viewed along the *a*-axis direction with N—H⋯O hydrogen bonds depicted by dashed lines. Hydrogen atoms not involved in these inter­actions are omitted for clarity.

In the crystal, inversion dimers are formed by pair-wise N1—H1*N*⋯O2^ii^ hydrogen bonds (Table 1 ▸) and these are connected into layers of mol­ecules parallel to the *bc* plane by N3—H3*N*⋯O3^i^ hydrogen bonds (Table 1 ▸ and Fig. 2 ▸). The layers pack along the *a*-axis direction with normal van der Waals contacts between layers.

## 4. Database survey

**Figure 3:**
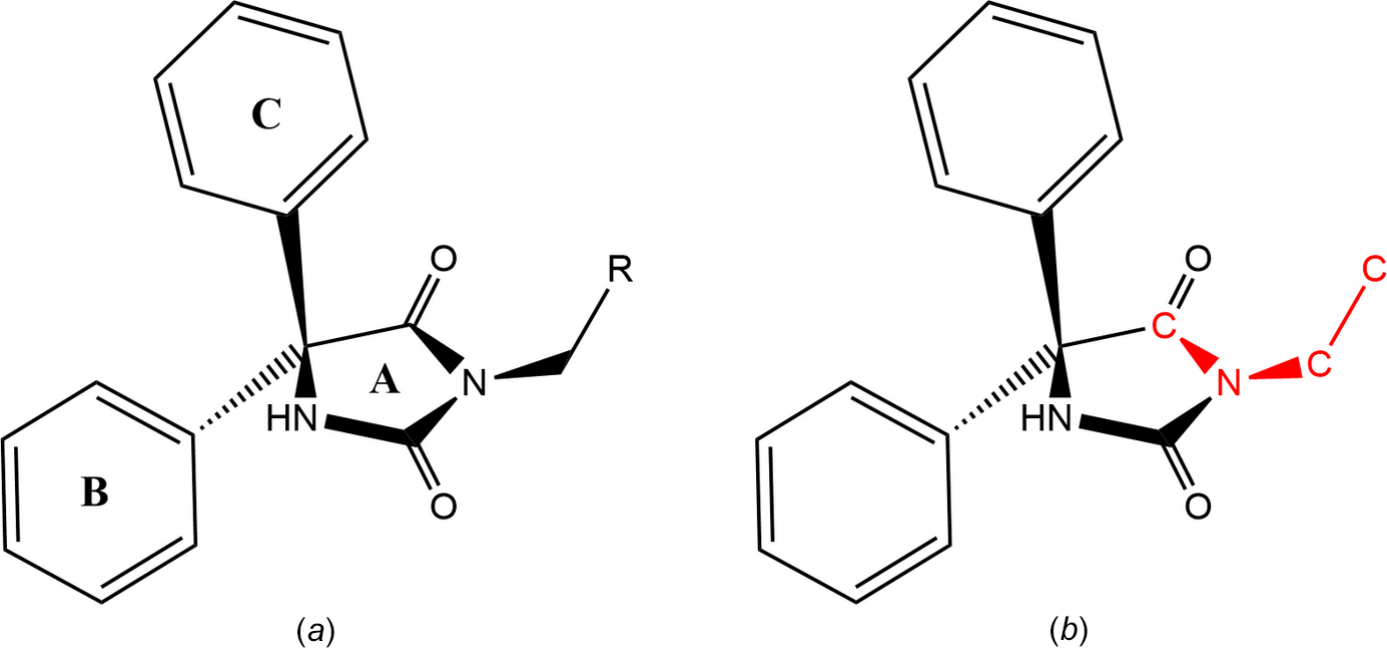
The search fragment (*R* = anything) used for the Database survey (**A**) and the key to Table 2 ▸ (**B**) with the relevant torsion angle highlighted in red.

A search of the Cambridge Structural Database (CSD, updated to April 2026; Groom *et al.*, 2016 ▸) with the search fragment shown in Fig. 3 ▸*a* (*R* = any atom or group) yielded eighteen hits, which are listed in Table 2 ▸, together with the most salient geometrical parameters. These are the dihedral angles between the imidazolidine ring and the attached phenyl groups and the torsion angle associated with the substituent on the ring N atom. In most instances, the imidazolidine ring is planar within experimental error but in CSD refcode JALGEL (Ramli *et al.*, 2017 ▸) and in one of the two independent mol­ecules in GITSOT (Mague *et al.*, 2014 ▸), GITSOT01 (Alanazi *et al.*, 2013 ▸) and QENBOD (Guerrab *et al.*, 2018*c* ▸), these rings are sufficiently non-planar that Cremer–Pople puckering parameters can be obtained. These are *Q*(2) = 0.0712 (16) Å and φ(2) = 279.3 (13)° in JALGEL, *Q*(2) = 0.0837 (3) Å and φ(2) = 156 (3)° in GITSOT, *Q*(2) = 0.080 (2) Å and φ(2) = 331.1 (11)° in GITSOT01 and *Q*(2) = 0.0829 (19) Å and φ(2) = 76.6 (13)° in QENBOD. The extent of puckering as measured by *Q*(2) is about the same for **3** as it is for the others cited above. The dihedral angles between the mean plane of the imidazolidine ring and those of the attached phenyl rings (Table 2 ▸) vary widely and are likely determined by a combination of intra­molecular inter­actions and packing considerations. The torsion angle associated with the ‘root’ of the –CH_2_*R* substituent (Fig. 3 ▸*b* and Table 2 ▸), while having a fairly large range because of the different sizes of the *R* group, is, nevertheless, much closer to 90° than to 0°, indicating that the group is well out of the plane of the imidazolidine ring and thus is *syn* to one of the phenyl groups (**C** in Fig. 3 ▸*a*) attached to that ring.

## 5. Hirshfeld surface analysis

**Figure 4:**
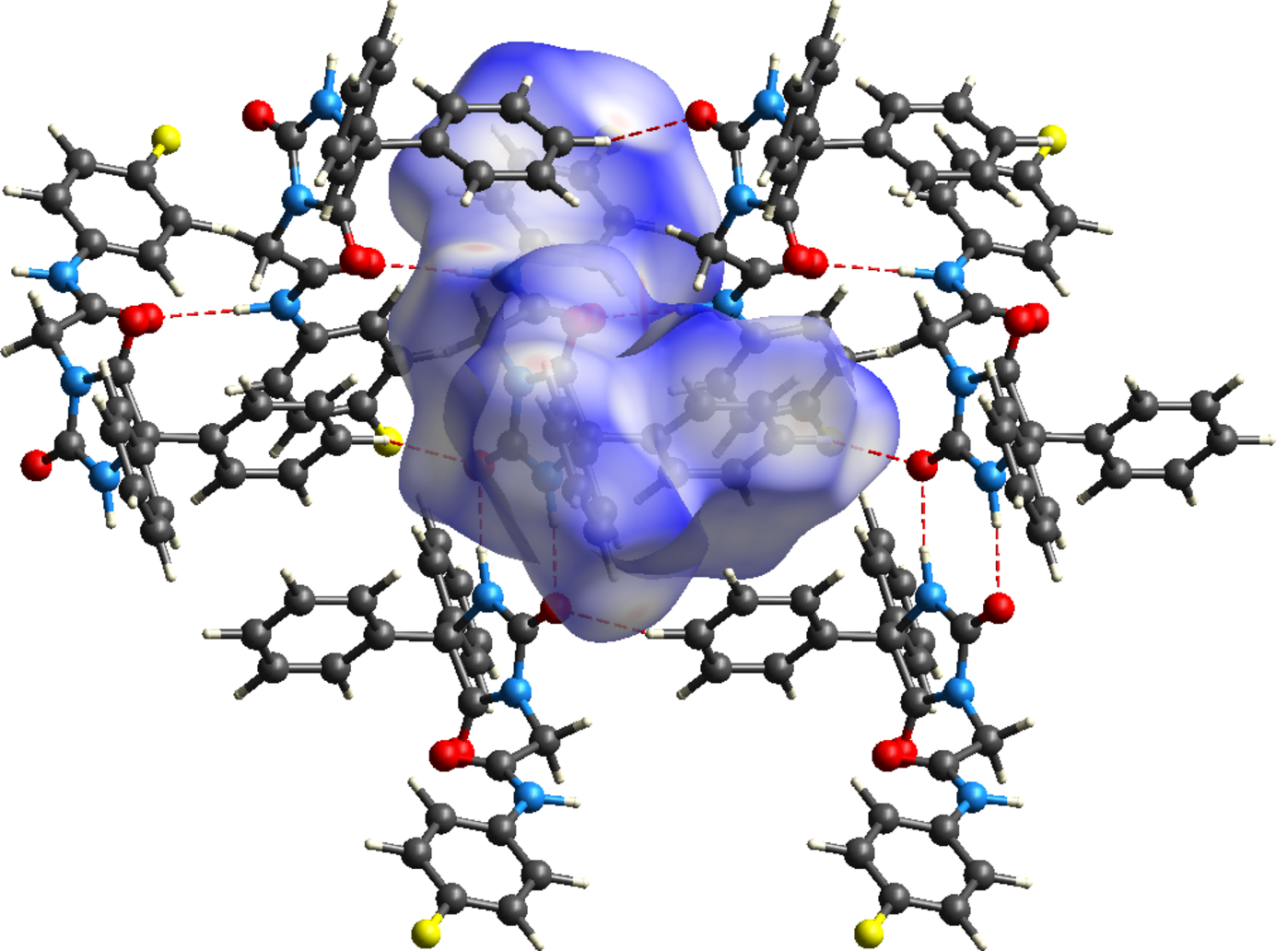
The *d*_norm_ surface for **3** with several neighboring mol­ecules of one layer in the crystal packing. The N—H⋯O hydrogen bonds are depicted by dashed lines.

**Figure 5:**
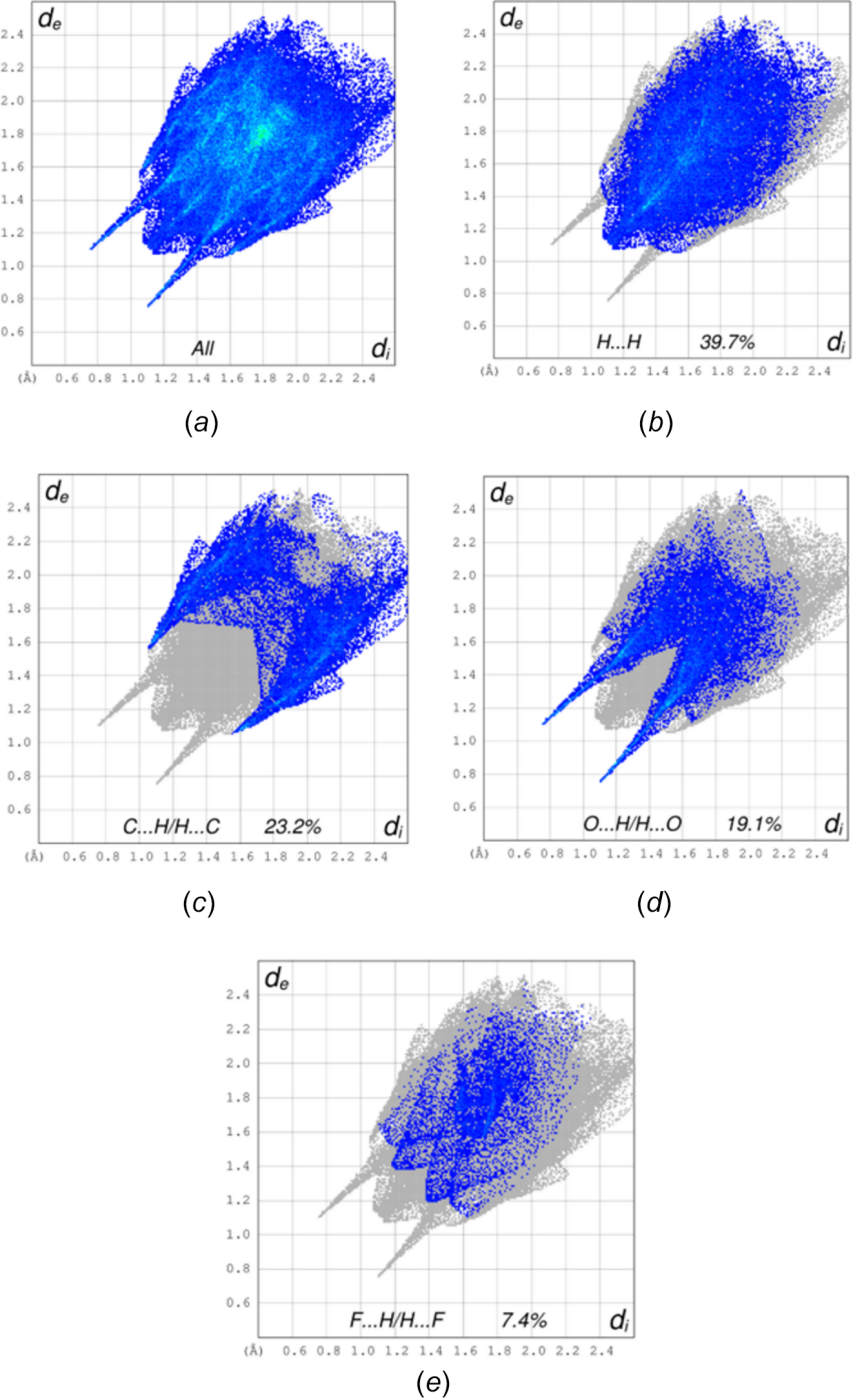
Selected two-dimensional fingerprint plots for **3** showing (*a*) all inter­molecular inter­actions and those delineated into (*b*) H⋯H, (*c*) C⋯H/H⋯C, (*d*) O⋯H/H⋯O and (*e*) F⋯H/H⋯F inter­actions.

The Hirshfeld surface analysis was carried out with *CrystalExplorer* (Spackman *et al.*, 2021 ▸); descriptions and inter­pretations of the plots obtained have been published previously (Tan *et al.*, 2019 ▸). Fig. 4 ▸ presents the *d*_norm_ surface for **3** together with several neighboring mol­ecules viewed along the *a*-axis direction, thus giving a rendition comparable to that in Fig. 2 ▸. The several N—H⋯O hydrogen bonds forming the layer penetrate the surface at the red spots. The two-dimensional fingerprint plots are shown in Fig. 5 ▸ with all inter­molecular inter­actions shown in Fig. 5 ▸*a*, while delineations into specific types of contacts appear in Fig. 5 ▸*b–e*. As is frequently the case, the H⋯H contacts comprise the largest fraction of the inter­molecular inter­actions (39.7%) since the periphery of the mol­ecule consists of hydrogen atoms. However, it is a smaller fraction than in many other cases, since the mol­ecule is not globular in shape. It is somewhat surprising that the C⋯H/H⋯C inter­actions show a quite high contribution (23.2%), as there are no significant C—H⋯π(ring) inter­actions; however, perusal of the inter­molecular C⋯H distances shows that there are eleven which are less than, or only slightly larger than, the sum of their van der Waals radii. The O⋯H/H⋯O contacts appear as a pair of sharp spikes at *d*_e_ + *d*_i_ ≃ 1.9 Å and can be attributed to the N—H⋯O hydrogen bonds. The only other significant contribution comes from the F⋯H/H⋯F contacts (Fig. 5 ▸*e*), which appear as two pairs of very broad peaks indicating a moderate range of F⋯H distances. These result from inter­actions between layers of mol­ecules, since the F atoms extend outward from the top and bottom of the layers. All other atom⋯atom contacts contribute less than 4% each.

## 6. Synthesis and crystallization

**Figure 6:**
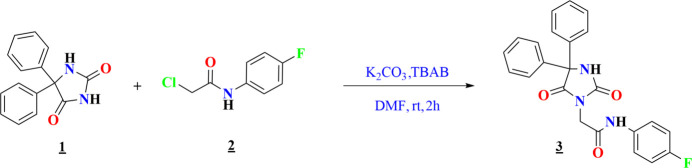
Reaction scheme for the synthesis of 2-(2,5-dioxo-4,4-di­phenyl­imidazolidin-1-yl)-*N*-(4-fluoro­phen­yl)acetamide (**3**).

The reaction scheme is shown in Fig. 6 ▸. Phenytoin (0.5 g, 1.98 mmol) and potassium carbonate (0.27 g, 1.95 mmol) were dissolved in di­methyl­formamide (10 mL), to which was added 2-chloro-*N*-(4-fluoro­phen­yl)acetamide (1.98 mmol) along with a catalytic amount of TBAB (tributyl ammonium bromide). Under reflux, the reaction was stirred for 2 h at 355 K. When the starting reagents had reacted completely, distilled water (100 ml) was added. The product precipitated in solid form, was filtered, dried and recrystallized from ethanol solution to afford colorless blocks.

Yield = 91.25%; color: white; m.p. = 510–512 K. FT–IR (ATR, cm^−1^): 3214 (N—H_amide_), 2937 (C–H_aliphatic_), 1692 (C=O). ^1^H NMR (500 MHz, DMSO-*d*_6_) ppm: 4.33 (*s*, 2H, CH_2_), 7.16–7.64 (*m*, 14H, H—Ar), 9.75 (*s*, 1H, NH_lactam_), 10.47 (*s*, 1H, NH_amide_). ^13^C NMR (125 MHz, DMSO-*d*_6_) ppm: 40.10 (CH_2_), 115.41–128.46 (CH_Ar_), 134.89 (Cq), 134–139 (Cq _Ar_), 155.73 (C=O),164.50 (C=O_ester_). HRMS (ESI): calculated for C_23_H_18_FN_3_O_3_ [*M* + H]^+^: 404.400; found 404.140.

## 7. Refinement

Crystal data, data collection and structure refinement details are summarized in Table 3 ▸. The carbon-bound H atoms were placed in calculated positions and refined isotropically using the riding model, with C—H distances ranging from 0.95 to 0.99 Å and *U*_iso_(H) set to 1.2 *U*_eq_(C). The N-bound hydrogen atoms H1*N* and H3*N* were located in difference-Fourier maps and refined freely.

## Supplementary Material

Crystal structure: contains datablock(s) I. DOI: 10.1107/S2056989026006201/vm2332sup1.cif

Structure factors: contains datablock(s) I. DOI: 10.1107/S2056989026006201/vm2332Isup2.hkl

Supporting information file. DOI: 10.1107/S2056989026006201/vm2332Isup3.cml

CCDC reference: 2562255

Additional supporting information:  crystallographic information; 3D view; checkCIF report

## Figures and Tables

**Table 1:** Hydrogen-bond geometry (Å, °)

*D*—H⋯*A*	*D*—H	H⋯*A*	*D*⋯*A*	*D*—H⋯*A*
C19—H19⋯O3	0.95	2.33	2.894 (2)	118
N1—H1*N*⋯O2^i^	0.90 (1)	1.96 (1)	2.857 (1)	172 (1)
N3—H3*N*⋯O3^ii^	0.89 (1)	2.08 (1)	2.959 (1)	168 (1)

Symmetry codes: (i) 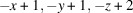; (ii) 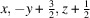.

**Table 2:** Database survey results

Compound	*R*	Dihedral angles (°)^*a*^	Torsion angle (°)^*b*^	Reference
3	C(=O)NH(4-FC_6_H_4_)	54.44 (7), 71.84 (7)	77.06 (14)	This work
BUCDEL	CH=CH_2_	64.55 (12), 62.07 (13)	96.4 (3)	Guerrab *et al.* (2020*a* ▸)
EKANOT	CH(OH)CH_2_N[(CH_2_)_2_]_2_NPh	60.57 (8), 84.91 (8)	83.75 (14)	Kieć-Kononowicz *et al.* (2003 ▸)
FEHPUG	Me	63.04 (5), 64.03 (5)	95.91 (12)	Guerrab *et al.* (2017*a* ▸)
GEMSOJ	*n*-Bu	70.38 (12), 63.85 (12)	83.70 (14)	Guerrab *et al.* (2017*b* ▸)
GITSOT	C(=O)(4-FC_6_H_4_)	60.56 (16), 82.66 (16); 66.36 (16), 84.94 (16)	−92.3 (3); 88.8 (3)	Mague *et al.* (2014 ▸)
GITSOT01	C(=O)(4-FC_6_H_4_)	61.58 (13), 81.17 (13); 66.36 (16), 84.94 (16)	−90.3 (3); −87.6 (3)	Alanazi *et al.* (2013 ▸)
JALGEL	COOEt	61.80 (9), 86.58 (16)	−70.7 (2)	Ramli *et al.* (2017 ▸)
LOKXAO	CH_2_N[(CH_2_)_2_]_2_O	76.55 (7), 68.07 (7)	103.66 (14)	Lamssane *et al.* (2024 ▸)
MESSAH	Ph	72.22 (7), 71.62 (6); 77.25 (7), 70.22 (6)	−95.07 (13); −87.88 (13)	Guerrab *et al.* (2018*a* ▸)
NIBMOE	CH_2_Br	63.60 (16), 76.45 (16)	−113.9 (3)	Guerrab *et al.* (2023 ▸)
PAJMAS	*n*-non­yl	54.03 (7), 60.67 (7)	106.90 (14)	Guerrab *et al.* (2022*a* ▸)
PEPDUM	H	59.17 (6), 53.21 (6)	–	Guerrab *et al.* (2017*c* ▸)
QAGPAT	*n*-oct­yl	76.05 (11), 63.46 (11)	89.55 (18)	Guerrab *et al.* (2020*b* ▸)
QENBET	*n*-prop­yl	58.08 (6), 66.31 (5)	79.83 (11)	Guerrab *et al.* (2018*b* ▸)
QENBOD	*n*-pent­yl	71.80 (12), 69.71 (12); 67.85 (10), 71.24 (11)	77.5 (3); −65.2 (3)	Guerrab *et al.* (2018*c* ▸)
WEMQUD	Et	64.48 (6), 71.25 (6); 66.09 (6), 67.13 (6)	76.00 (14); 113.95 (13)	Guerrab *et al.* (2017*d* ▸)
WEMQUD01	Et	64.649 (10), 69.34 (10)	−68.2 (3)	Trišović *et al.* (2019 ▸)
YEDYOZ	*i*-prop­yl	73.04 (5), 68.42 (5)	72.65 (11)	Guerrab *et al.* (2022*b* ▸)

Notes: (*a*) Dihedral angles between the mean plane of ring A and those of rings B and C, respectively, as defined in Fig. 3 ▸*b*. Where two pairs of values occur, these refer to independent mol­ecules in the asymmetric unit. (*b*) The C—N—C—C torsion angle as defined in Fig. 3 ▸*b*. Where two values occur, these refer to independent mol­ecules in the asymmetric unit.

**Table 3:** Experimental details

Crystal data	
Chemical formula	C_23_H_18_FN_3_O_3_
*M*_r_	403.40
Crystal system, space group	Monoclinic, *P*2_1_/*c*
Temperature (K)	200
*a*, *b*, *c* (Å)	9.583 (3), 21.151 (5), 9.825 (2)
β (°)	104.355 (9)
*V* (Å^3^)	1929.2 (9)
*Z*	4
Radiation type	Cu *K*α
μ (mm^−1^)	0.83
Crystal size (mm)	0.14 × 0.12 × 0.09

Data collection	
Diffractometer	Bruker D8 Venture PhotonII
Absorption correction	Multi-scan (*SADABS*; Krause *et al.*, 2015 ▸)
*T*_min_, *T*_max_	0.610, 0.753
No. of measured, independent and observed [*I* > 2σ(*I*)] reflections	16290, 3527, 3340
*R*_int_	0.043
(sin θ/λ)_max_ (Å^−1^)	0.604

Refinement	
*R*[*F*^2^ > 2σ(*F*^2^)], *wR*(*F*^2^), *S*	0.038, 0.102, 1.04
No. of reflections	3527
No. of parameters	280
No. of restraints	2
H-atom treatment	H atoms treated by a mixture of independent and constrained refinement
Δρ_max_, Δρ_min_ (e Å^−3^)	0.22, −0.20

Computer programs: *APEX3* and *SAINT* (Bruker, 2016 ▸), *SHELXT2018/2* (Sheldrick, 2015*a* ▸), *SHELXL2019/3* (Sheldrick, 2015*b* ▸), *DIAMOND* (Brandenburg & Putz, 2012 ▸) and *SHELXTL* (Sheldrick, 2008 ▸).

## supplementary crystallographic information

### 2-(2,5-Dioxo-4,4-diphenylimidazolidin-1-yl)-N-(4-fluorophenyl)acetamide Crystal data

**Table:**

C_23_H_18_FN_3_O_3_	*F*(000) = 840
*M**_r_* = 403.40	*D*_x_ = 1.389 Mg m^−^^3^
Monoclinic, *P*2_1_/*c*	Cu *K*α radiation, λ = 1.54178 Å
*a* = 9.583 (3) Å	Cell parameters from 745 reflections
*b* = 21.151 (5) Å	θ = 4.2–68.5°
*c* = 9.825 (2) Å	µ = 0.83 mm^−^^1^
β = 104.355 (9)°	*T* = 200 K
*V* = 1929.2 (9) Å^3^	Prismatic, colourless
*Z* = 4	0.14 × 0.12 × 0.09 mm

### 2-(2,5-Dioxo-4,4-diphenylimidazolidin-1-yl)-N-(4-fluorophenyl)acetamide Data collection

**Table:**

Bruker D8 Venture PhotonII diffractometer	3527 independent reflections
Radiation source: fine-focus sealed tube	3340 reflections with *I* > 2σ(*I*)
Graphite monochromator	*R*_int_ = 0.043
phi & ω scan	θ_max_ = 68.5°, θ_min_ = 4.2°
Absorption correction: multi-scan (SADABS; Krause *et al.*, 2015)	*h* = −11→11
*T*_min_ = 0.610, *T*_max_ = 0.753	*k* = −25→25
16290 measured reflections	*l* = −10→11

### 2-(2,5-Dioxo-4,4-diphenylimidazolidin-1-yl)-N-(4-fluorophenyl)acetamide Refinement

**Table:**

Refinement on *F*^2^	Secondary atom site location: difference Fourier map
Least-squares matrix: full	Hydrogen site location: mixed
*R*[*F*^2^ > 2σ(*F*^2^)] = 0.038	H atoms treated by a mixture of independent and constrained refinement
*wR*(*F*^2^) = 0.102	*w* = 1/[σ^2^(*F*_o_^2^) + (0.0521*P*)^2^ + 0.494*P*] where *P* = (*F*_o_^2^ + 2*F*_c_^2^)/3
*S* = 1.04	(Δ/σ)_max_ = 0.001
3527 reflections	Δρ_max_ = 0.22 e Å^−^^3^
280 parameters	Δρ_min_ = −0.20 e Å^−^^3^
2 restraints	Extinction correction: *SHELXL-2019/3* (Lübben *et al.*, 2015b), Fc^*^=kFc[1+0.001xFc^2^λ^3^/sin(2θ)]^-1/4^
Primary atom site location: dual	Extinction coefficient: 0.0059 (5)

### 2-(2,5-Dioxo-4,4-diphenylimidazolidin-1-yl)-N-(4-fluorophenyl)acetamide Special details

**Table:**

Geometry. All esds (except the esd in the dihedral angle between two l.s. planes) are estimated using the full covariance matrix. The cell esds are taken into account individually in the estimation of esds in distances, angles and torsion angles; correlations between esds in cell parameters are only used when they are defined by crystal symmetry. An approximate (isotropic) treatment of cell esds is used for estimating esds involving l.s. planes.
Refinement. Refinement of F^2^ against ALL reflections. The weighted R-factor wR and goodness of fit S are based on F^2^, conventional R-factors R are based on F, with F set to zero for negative F^2^. The threshold expression of F^2^ > 2sigma(F^2^) is used only for calculating R-factors(gt) etc. and is not relevant to the choice of reflections for refinement. R-factors based on F^2^ are statistically about twice as large as those based on F, and R- factors based on ALL data will be even larger. H-atoms attached to carbon were placed in calculated positions (C—H = 0.95 - 0.99 Å) and were included as riding contributions with isotropic displacement parameters 1.2 - 1.5 times those of the attached atoms. Those attached to nitrogen were placed in locations derived from a difference map and refined with DFIX 0.91 0.01 instructions

### 2-(2,5-Dioxo-4,4-diphenylimidazolidin-1-yl)-N-(4-fluorophenyl)acetamide Fractional atomic coordinates and isotropic or equivalent isotropic displacement parameters (Å^2^)

**Table:**

	*x*	*y*	*z*	*U*_iso_*/*U*_eq_	
N1	0.59592 (11)	0.56102 (5)	0.93246 (10)	0.0294 (2)	
N2	0.55093 (11)	0.65769 (4)	0.99651 (10)	0.0293 (2)	
N3	0.26313 (11)	0.76434 (5)	1.02613 (10)	0.0329 (2)	
O1	0.66903 (11)	0.71876 (4)	0.86682 (10)	0.0399 (2)	
O2	0.45097 (10)	0.57242 (4)	1.08624 (9)	0.0343 (2)	
O3	0.32384 (10)	0.72223 (4)	0.83500 (8)	0.0389 (2)	
F1	−0.22620 (11)	0.90330 (7)	0.79317 (13)	0.0827 (4)	
C1	0.63048 (13)	0.66793 (5)	0.90015 (12)	0.0294 (3)	
C2	0.52504 (12)	0.59313 (5)	1.01160 (11)	0.0273 (3)	
C3	0.65601 (13)	0.60209 (5)	0.84200 (12)	0.0281 (3)	
C4	0.56788 (13)	0.59940 (5)	0.68856 (12)	0.0290 (3)	
C5	0.61639 (14)	0.63309 (6)	0.58776 (13)	0.0351 (3)	
H5	0.703801	0.656307	0.614259	0.042*	
C6	0.53795 (16)	0.63295 (6)	0.44892 (14)	0.0417 (3)	
H6	0.570978	0.656650	0.380929	0.050*	
C7	0.41212 (16)	0.59860 (7)	0.40885 (14)	0.0443 (3)	
H7	0.358480	0.598570	0.313532	0.053*	
C8	0.36454 (16)	0.56424 (7)	0.50807 (15)	0.0469 (3)	
H8	0.278588	0.540073	0.480525	0.056*	
C9	0.44159 (15)	0.56479 (7)	0.64792 (14)	0.0392 (3)	
H9	0.407649	0.541407	0.715790	0.047*	
C10	0.81371 (13)	0.58587 (6)	0.85289 (12)	0.0302 (3)	
C11	0.84269 (15)	0.52560 (7)	0.81017 (14)	0.0408 (3)	
H11	0.765407	0.496999	0.776413	0.049*	
C12	0.98215 (17)	0.50666 (8)	0.81608 (16)	0.0501 (4)	
H12	1.000474	0.465387	0.786526	0.060*	
C13	1.09465 (16)	0.54816 (9)	0.86523 (17)	0.0532 (4)	
H13	1.190757	0.535595	0.869216	0.064*	
C14	1.06728 (16)	0.60748 (8)	0.90821 (19)	0.0563 (4)	
H14	1.145121	0.635690	0.942874	0.068*	
C15	0.92694 (15)	0.62706 (7)	0.90184 (16)	0.0437 (3)	
H15	0.909242	0.668487	0.930978	0.052*	
C16	0.48547 (13)	0.70686 (5)	1.06182 (12)	0.0311 (3)	
H16A	0.554874	0.741958	1.090658	0.037*	
H16B	0.461651	0.689884	1.147225	0.037*	
C17	0.34876 (13)	0.73197 (5)	0.96152 (12)	0.0288 (3)	
C18	0.13455 (13)	0.79778 (6)	0.96256 (13)	0.0343 (3)	
C19	0.05425 (16)	0.78569 (8)	0.82658 (15)	0.0493 (4)	
H19	0.083336	0.753542	0.771945	0.059*	
C20	−0.06887 (17)	0.82104 (10)	0.77136 (17)	0.0598 (4)	
H20	−0.124894	0.813180	0.678686	0.072*	
C21	−0.10874 (16)	0.86712 (9)	0.85115 (18)	0.0556 (4)	
C22	−0.03462 (17)	0.87877 (8)	0.98676 (18)	0.0542 (4)	
H22	−0.066397	0.910180	1.041330	0.065*	
C23	0.08855 (15)	0.84336 (7)	1.04252 (15)	0.0427 (3)	
H23	0.141664	0.850554	1.136552	0.051*	
H3N	0.2951 (15)	0.7691 (7)	1.1191 (10)	0.037 (4)*	
H1N	0.5852 (16)	0.5190 (4)	0.9197 (15)	0.040 (4)*	

### 2-(2,5-Dioxo-4,4-diphenylimidazolidin-1-yl)-N-(4-fluorophenyl)acetamide Atomic displacement parameters (Å^2^)

**Table:**

	*U*^11^	*U*^22^	*U*^33^	*U*^12^	*U*^13^	*U*^23^
N1	0.0402 (6)	0.0224 (5)	0.0288 (5)	−0.0026 (4)	0.0147 (4)	0.0005 (4)
N2	0.0386 (5)	0.0247 (5)	0.0259 (5)	−0.0008 (4)	0.0108 (4)	−0.0011 (4)
N3	0.0385 (6)	0.0382 (6)	0.0206 (5)	0.0041 (4)	0.0051 (4)	−0.0012 (4)
O1	0.0535 (6)	0.0260 (4)	0.0447 (5)	−0.0050 (4)	0.0208 (4)	0.0012 (4)
O2	0.0436 (5)	0.0319 (4)	0.0317 (4)	−0.0037 (4)	0.0178 (4)	−0.0001 (3)
O3	0.0448 (5)	0.0499 (5)	0.0212 (4)	0.0000 (4)	0.0069 (4)	−0.0045 (4)
F1	0.0465 (6)	0.1175 (10)	0.0852 (8)	0.0338 (6)	0.0184 (5)	0.0446 (7)
C1	0.0353 (6)	0.0269 (6)	0.0259 (5)	−0.0017 (4)	0.0076 (5)	0.0003 (4)
C2	0.0317 (6)	0.0270 (6)	0.0223 (5)	−0.0017 (4)	0.0047 (4)	0.0004 (4)
C3	0.0350 (6)	0.0245 (6)	0.0269 (6)	−0.0026 (4)	0.0114 (5)	0.0011 (4)
C4	0.0336 (6)	0.0272 (6)	0.0276 (6)	0.0038 (4)	0.0103 (5)	−0.0017 (4)
C5	0.0400 (7)	0.0338 (6)	0.0330 (6)	0.0023 (5)	0.0123 (5)	0.0039 (5)
C6	0.0551 (8)	0.0413 (7)	0.0303 (6)	0.0084 (6)	0.0136 (6)	0.0059 (5)
C7	0.0534 (8)	0.0481 (8)	0.0280 (6)	0.0085 (6)	0.0036 (6)	−0.0055 (5)
C8	0.0442 (8)	0.0553 (9)	0.0384 (7)	−0.0066 (6)	0.0050 (6)	−0.0104 (6)
C9	0.0420 (7)	0.0440 (7)	0.0329 (6)	−0.0069 (5)	0.0120 (5)	−0.0032 (5)
C10	0.0339 (6)	0.0331 (6)	0.0242 (5)	0.0001 (5)	0.0085 (5)	0.0042 (4)
C11	0.0393 (7)	0.0404 (7)	0.0417 (7)	0.0036 (5)	0.0085 (6)	−0.0046 (6)
C12	0.0489 (8)	0.0540 (9)	0.0486 (8)	0.0167 (7)	0.0143 (7)	0.0020 (7)
C13	0.0370 (7)	0.0713 (11)	0.0535 (9)	0.0098 (7)	0.0154 (6)	0.0191 (8)
C14	0.0370 (8)	0.0626 (10)	0.0686 (10)	−0.0095 (7)	0.0115 (7)	0.0096 (8)
C15	0.0404 (7)	0.0402 (7)	0.0512 (8)	−0.0073 (6)	0.0126 (6)	0.0010 (6)
C16	0.0402 (7)	0.0292 (6)	0.0234 (5)	0.0018 (5)	0.0068 (5)	−0.0039 (4)
C17	0.0373 (6)	0.0270 (6)	0.0227 (6)	−0.0046 (5)	0.0084 (5)	−0.0011 (4)
C18	0.0343 (6)	0.0398 (7)	0.0295 (6)	0.0002 (5)	0.0091 (5)	0.0054 (5)
C19	0.0445 (8)	0.0668 (10)	0.0337 (7)	0.0048 (7)	0.0041 (6)	0.0004 (6)
C20	0.0420 (8)	0.0949 (13)	0.0386 (8)	0.0055 (8)	0.0024 (6)	0.0153 (8)
C21	0.0348 (7)	0.0773 (11)	0.0569 (9)	0.0136 (7)	0.0152 (7)	0.0293 (8)
C22	0.0470 (8)	0.0601 (9)	0.0605 (10)	0.0145 (7)	0.0229 (7)	0.0091 (7)
C23	0.0408 (7)	0.0502 (8)	0.0383 (7)	0.0063 (6)	0.0122 (6)	0.0020 (6)

### 2-(2,5-Dioxo-4,4-diphenylimidazolidin-1-yl)-N-(4-fluorophenyl)acetamide Geometric parameters (Å, º)

**Table:**

N1—C2	1.3375 (15)	C9—H9	0.9500
N1—C3	1.4594 (14)	C10—C15	1.3817 (19)
N1—H1N	0.899 (9)	C10—C11	1.3915 (18)
N2—C1	1.3719 (15)	C11—C12	1.383 (2)
N2—C2	1.4023 (15)	C11—H11	0.9500
N2—C16	1.4445 (15)	C12—C13	1.381 (2)
N3—C17	1.3428 (16)	C12—H12	0.9500
N3—C18	1.4238 (16)	C13—C14	1.370 (3)
N3—H3N	0.895 (9)	C13—H13	0.9500
O1—C1	1.2081 (15)	C14—C15	1.394 (2)
O2—C2	1.2213 (14)	C14—H14	0.9500
O3—C17	1.2237 (14)	C15—H15	0.9500
F1—C21	1.3635 (18)	C16—C17	1.5263 (17)
C1—C3	1.5477 (16)	C16—H16A	0.9900
C3—C10	1.5273 (17)	C16—H16B	0.9900
C3—C4	1.5355 (16)	C18—C23	1.3829 (19)
C4—C9	1.3859 (18)	C18—C19	1.3902 (19)
C4—C5	1.3903 (17)	C19—C20	1.388 (2)
C5—C6	1.3846 (19)	C19—H19	0.9500
C5—H5	0.9500	C20—C21	1.363 (3)
C6—C7	1.379 (2)	C20—H20	0.9500
C6—H6	0.9500	C21—C22	1.368 (3)
C7—C8	1.381 (2)	C22—C23	1.391 (2)
C7—H7	0.9500	C22—H22	0.9500
C8—C9	1.389 (2)	C23—H23	0.9500
C8—H8	0.9500		
			
C2—N1—C3	112.69 (9)	C12—C11—C10	121.05 (13)
C2—N1—H1N	121.7 (10)	C12—C11—H11	119.5
C3—N1—H1N	123.5 (10)	C10—C11—H11	119.5
C1—N2—C2	111.71 (9)	C13—C12—C11	119.55 (14)
C1—N2—C16	124.72 (10)	C13—C12—H12	120.2
C2—N2—C16	123.13 (10)	C11—C12—H12	120.2
C17—N3—C18	127.57 (10)	C14—C13—C12	119.83 (14)
C17—N3—H3N	116.5 (10)	C14—C13—H13	120.1
C18—N3—H3N	115.6 (10)	C12—C13—H13	120.1
O1—C1—N2	125.96 (11)	C13—C14—C15	120.98 (15)
O1—C1—C3	127.86 (10)	C13—C14—H14	119.5
N2—C1—C3	106.16 (9)	C15—C14—H14	119.5
O2—C2—N1	128.42 (11)	C10—C15—C14	119.61 (14)
O2—C2—N2	123.86 (10)	C10—C15—H15	120.2
N1—C2—N2	107.72 (9)	C14—C15—H15	120.2
N1—C3—C10	110.89 (9)	N2—C16—C17	111.19 (9)
N1—C3—C4	111.83 (9)	N2—C16—H16A	109.4
C10—C3—C4	110.75 (9)	C17—C16—H16A	109.4
N1—C3—C1	100.86 (9)	N2—C16—H16B	109.4
C10—C3—C1	114.87 (10)	C17—C16—H16B	109.4
C4—C3—C1	107.27 (9)	H16A—C16—H16B	108.0
C9—C4—C5	119.20 (11)	O3—C17—N3	125.30 (11)
C9—C4—C3	122.07 (10)	O3—C17—C16	121.16 (11)
C5—C4—C3	118.73 (11)	N3—C17—C16	113.55 (10)
C6—C5—C4	120.33 (12)	C23—C18—C19	119.71 (13)
C6—C5—H5	119.8	C23—C18—N3	117.41 (11)
C4—C5—H5	119.8	C19—C18—N3	122.87 (12)
C7—C6—C5	120.34 (12)	C20—C19—C18	119.39 (15)
C7—C6—H6	119.8	C20—C19—H19	120.3
C5—C6—H6	119.8	C18—C19—H19	120.3
C6—C7—C8	119.62 (13)	C21—C20—C19	119.50 (15)
C6—C7—H7	120.2	C21—C20—H20	120.3
C8—C7—H7	120.2	C19—C20—H20	120.3
C7—C8—C9	120.42 (13)	C20—C21—F1	118.75 (16)
C7—C8—H8	119.8	C20—C21—C22	122.47 (14)
C9—C8—H8	119.8	F1—C21—C22	118.78 (16)
C4—C9—C8	120.09 (12)	C21—C22—C23	118.15 (15)
C4—C9—H9	120.0	C21—C22—H22	120.9
C8—C9—H9	120.0	C23—C22—H22	120.9
C15—C10—C11	118.98 (12)	C18—C23—C22	120.71 (14)
C15—C10—C3	124.11 (11)	C18—C23—H23	119.6
C11—C10—C3	116.91 (11)	C22—C23—H23	119.6
			
C2—N2—C1—O1	−179.64 (12)	N1—C3—C10—C15	−117.55 (13)
C16—N2—C1—O1	7.82 (19)	C4—C3—C10—C15	117.68 (13)
C2—N2—C1—C3	1.79 (13)	C1—C3—C10—C15	−4.02 (16)
C16—N2—C1—C3	−170.76 (10)	N1—C3—C10—C11	62.50 (13)
C3—N1—C2—O2	171.70 (11)	C4—C3—C10—C11	−62.27 (13)
C3—N1—C2—N2	−8.92 (13)	C1—C3—C10—C11	176.03 (10)
C1—N2—C2—O2	−176.40 (11)	C15—C10—C11—C12	0.0 (2)
C16—N2—C2—O2	−3.72 (17)	C3—C10—C11—C12	179.94 (12)
C1—N2—C2—N1	4.19 (13)	C10—C11—C12—C13	0.0 (2)
C16—N2—C2—N1	176.87 (10)	C11—C12—C13—C14	0.3 (2)
C2—N1—C3—C10	131.58 (10)	C12—C13—C14—C15	−0.7 (2)
C2—N1—C3—C4	−104.27 (11)	C11—C10—C15—C14	−0.3 (2)
C2—N1—C3—C1	9.47 (12)	C3—C10—C15—C14	179.70 (13)
O1—C1—C3—N1	175.05 (12)	C13—C14—C15—C10	0.7 (2)
N2—C1—C3—N1	−6.41 (11)	C1—N2—C16—C17	77.06 (14)
O1—C1—C3—C10	55.76 (16)	C2—N2—C16—C17	−94.66 (13)
N2—C1—C3—C10	−125.70 (10)	C18—N3—C17—O3	−5.5 (2)
O1—C1—C3—C4	−67.82 (16)	C18—N3—C17—C16	174.72 (11)
N2—C1—C3—C4	110.72 (10)	N2—C16—C17—O3	−17.08 (15)
N1—C3—C4—C9	4.11 (15)	N2—C16—C17—N3	162.72 (10)
C10—C3—C4—C9	128.35 (12)	C17—N3—C18—C23	−160.05 (12)
C1—C3—C4—C9	−105.58 (12)	C17—N3—C18—C19	21.0 (2)
N1—C3—C4—C5	−176.21 (10)	C23—C18—C19—C20	2.0 (2)
C10—C3—C4—C5	−51.97 (14)	N3—C18—C19—C20	−179.05 (14)
C1—C3—C4—C5	74.09 (13)	C18—C19—C20—C21	0.2 (2)
C9—C4—C5—C6	1.19 (18)	C19—C20—C21—F1	177.08 (15)
C3—C4—C5—C6	−178.49 (11)	C19—C20—C21—C22	−2.4 (3)
C4—C5—C6—C7	−1.04 (19)	C20—C21—C22—C23	2.2 (3)
C5—C6—C7—C8	0.0 (2)	F1—C21—C22—C23	−177.26 (14)
C6—C7—C8—C9	0.9 (2)	C19—C18—C23—C22	−2.2 (2)
C5—C4—C9—C8	−0.33 (19)	N3—C18—C23—C22	178.81 (13)
C3—C4—C9—C8	179.34 (12)	C21—C22—C23—C18	0.1 (2)
C7—C8—C9—C4	−0.7 (2)		

### 2-(2,5-Dioxo-4,4-diphenylimidazolidin-1-yl)-N-(4-fluorophenyl)acetamide Hydrogen-bond geometry (Å, º)

**Table:**

*D*—H···*A*	*D*—H	H···*A*	*D*···*A*	*D*—H···*A*
C19—H19···O3	0.95	2.33	2.894 (2)	118
N3—H3*N*···O3^i^	0.89 (1)	2.08 (1)	2.959 (1)	168 (1)
N1—H1*N*···O2^ii^	0.90 (1)	1.96 (1)	2.857 (1)	172 (1)

Symmetry codes: (i) *x*, −*y*+3/2, *z*+1/2; (ii) −*x*+1, −*y*+1, −*z*+2.
